# Complement System and Age-Related Macular Degeneration: Implications of Gene-Environment Interaction for Preventive and Personalized Medicine

**DOI:** 10.1155/2018/7532507

**Published:** 2018-08-26

**Authors:** Andrea Maugeri, Martina Barchitta, Maria Grazia Mazzone, Francesco Giuliano, Antonella Agodi

**Affiliations:** ^1^Department of Medical and Surgical Sciences and Advanced Technologies “GF Ingrassia”, University of Catania, Via S. Sofia 87, 95123 Catania, Italy; ^2^SIFI SpA, Research and Development Department, Via Ercole Patti 36, 95025 Catania, Italy

## Abstract

Age-related macular degeneration (AMD) is the most common cause of visual loss in developed countries, with a significant economic and social burden on public health. Although genome-wide and gene-candidate studies have been enabled to identify genetic variants in the complement system associated with AMD pathogenesis, the effect of gene-environment interaction is still under debate. In this review we provide an overview of the role of complement system and its genetic variants in AMD, summarizing the consequences of the interaction between genetic and environmental risk factors on AMD onset, progression, and therapeutic response. Finally, we discuss the perspectives of current evidence in the field of genomics driven personalized medicine and public health.

## 1. Introduction

Age-related macular degeneration (AMD), characterized by the progressive destruction of neurosensory retina at the macular area, is the most common cause of visual loss in developed countries, with a significant economic and social burden on public health [1]. The early stage of AMD leads to aberrant pigmentation of retinal pigment epithelium (RPE) and accumulation of extracellular material, called “drusen,” underneath the RPE basement membrane. Drusen are small, yellowish, extracellular deposits of lipid, cellular debris and protein that may lead to impaired RPE function and disruption of the metabolic transport between RPE and choroid [2]. The advanced stages manifest as choroidal neovascularization (CNV) in the wet AMD, or geographic atrophy (GA) in the dry AMD [3]. Pathological features of AMD are caused by the interaction of oxidative stress, impaired RPE activity and function, increased apoptosis, and abnormal immune system activation [4, 5]. Smoking is the strongest modifiable risk factor for AMD, leading to oxidative stress, ischemia, hypoxia, and neovascularization [6]. Although both current and former smoking may increase AMD risk, a protective effect has been observed for time since smoking cessation [7]. Particularly, subjects who had stopped smoking for more than 20 years were not at risk of advanced stages of AMD [8, 9]. Other modifiable risk factors, such as obesity [10–13] and sunlight exposure [14, 15], are still under debate, since their role in AMD susceptibility may be related to an overall unhealthy lifestyle [16–18]. To date, the only factor that may be protective against AMD is a healthy diet, rich in omega-3 fatty acids, lutein, zeaxanthin, and antioxidants [19–22]. Consistently, the Age-Related Eye Disease Study 2 (AREDS2) formulation (i.e., a combination of zinc, b-carotene, and vitamins C, and E) has been shown to reduce the risk of progression to advanced AMD [23]. While AREDS formulation represents the only available treatment for dry AMD, intravitreal injections of antivascular endothelial growth factor (VEGF) agents (i.e., ranibizumab, bevacizumab, and aflibercept) may improve visual acuity in patients with wet AMD [24–29].

In addition to the effect of modifiable factors, genetic variants confer about 60% of the attributable risk [30], with at least 34 genomic loci implicated in AMD pathogenesis [31]. Genetic risk factors associated with AMD susceptibility include polymorphisms in complement factor H (*CFH*) [32], age-related maculopathy susceptibility 2 (*ARMS2*) [33], apolipoprotein E (*APOE*) [34], and vascular endothelial growth factor (*VEGF)* [35]. Above all, the discovery of genetic variants in components of the complement system indicated the potential role of local inflammation and complement regulation in the pathogenesis of AMD [36].

Given this scenario, the perspective of personalized medicine for the prevention and treatment of AMD requires a more accurate evidence-based knowledge of gene-environment interactions.

Here we provide an overview of the role of complement system in AMD and summarize the consequences of the interaction between genetic and environmental risk factors on AMD onset and progression and therapeutic response. Finally, we discuss the perspectives of current evidence in the field of genomics driven personalized medicine.

## 2. The Complement System

**Figure 1:**
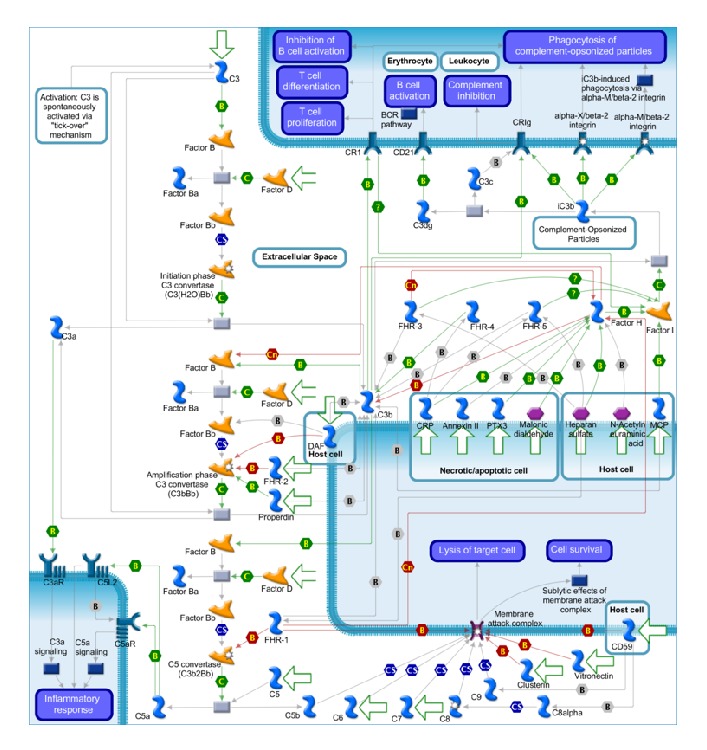
Complement system activation and regulation by the alternative pathway. This figure was prepared using MetaCore from Thomson Reuters.

The complement system is implicated in the innate immune response, which constitutes the first-line host defense against pathogenic infections [37]. It also functions as immunoregulatory system of clear immune complexes, inflammatory products, and apoptotic cells. Complement components constitute a complex network of about 30 plasma- and membrane-associated serum proteins, designated by numerals (C1-C9) or letter symbols (e.g., complement factors H, FH), which are organized into hierarchal proteolytic cascades. The activation of complement system involves three proteolytic cascades, namely, the classical, lectin, and alternative pathways, which lead to the activation of C3 convertase, the convergence point of all complement pathways. This downstream cascade is characterized by the activation of the following effectors: the membrane attack complex (MAC), anaphylatoxins (C3a and C5a), and opsonins. The first induces cell lysis, producing a pore-like structure in the phospholipid bilayer, and stimulates the release of anaphylatoxins and growth factors from the vascular endothelium. The classical and lectin pathways are, respectively, activated by binding to complement-fixing antibodies in immune complexes or to mannose residues on the surface of microorganisms. In contrast, the alternative pathway is spontaneously activated by a constant low-rate hydrolysis of C3, which further binds to factor B (FB), allowing factor D (FD) to cleave factor B into Ba and Bb. The resulting C3 convertase initiates the terminal pathway via an amplification loop, producing more C3b and C3a from C3 (Figure 1).

## 3. Regulation of Complement System

The refined balance between activation and inhibition of complement system is the crucial regulatory mechanism to prevent self-tissue damage [37, 38]. Although increased complement activity may be protective against chronic low-grade inflammation and infection in early life [39], lack of inhibition is associated with several diseases, such as systemic lupus erythematosus [40], atypical haemolytic uraemic syndrome [41], dense deposit disease [42], and AMD [43]. Therefore, complement system activity is strictly controlled by regulatory proteins, which mainly act by degrading complement components, increasing C3 convertase decay, and modulating the MAC assembly [44–46]. The first is a function of factor I (FI), which regulates the classical and alternative pathways by cleaving C3b into inactive fragments [47]. However, to prevent nonspecific degradation of complement components, the proteolytic activity of FI requires several cofactors, including complement receptor 1 (CR1), membrane cofactor protein (MCP), and FH [47–51], which accelerate C3 convertase decay by displacing factor Bb from existing C3 convertase [52, 53].

The ability of alternative pathway to discriminate between self and potential pathogens is conferred by recognition of glycosaminoglycans (GAGs) and sialic acid glycans (i.e., heparin-sulfate and N-acetylneuraminic acid) on host cells [54–57]. Binding of FH to the surface of necrotic cells and to apoptotic particles is mediated by CRP, Annexin II, DNA, and histones [58–61]. An additional complement inhibitor is the decay-accelerating factor (DAF), which inhibits assembly of neoformed C3 convertases and accelerates the decay of pre-existing convertases [46, 62–66]. Lastly, the regulation of complement system may be also provided by inhibiting MAC formation via membrane bound (CD59) or fluid-phase (Vitronectin and Clusterin) inhibitors [67–72].

## 4. Complement System and AMD Pathogenesis

**Figure 2:**
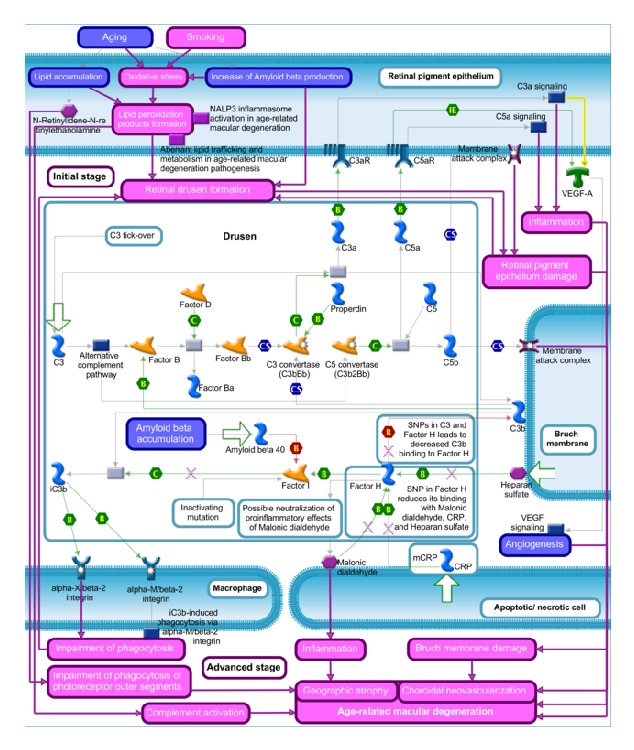
Complement system dysregulation in the age-related macular degeneration. This figure was prepared using MetaCore from Thomson Reuters.

Although the majority of circulating complement components is produced by the liver, the retina shows extrahepatic complement synthesis [73], probably to overcome the restricted access of plasma protein to the retina through the blood-retinal barrier. Several lines of evidence demonstrated that complement dysregulation, especially the alternative pathway, is involved in the pathogenesis of AMD. The major stressors for AMD development, such as aging, smoking, and oxidative stress, have been linked to the overactivation of the complement system (Figure 2). This evidence has been also supported by immune-histological and proteomic studies, which identified complement components as constituents of drusen, suggesting the local activation of the complement pathways [30, 74–77]. Increased levels of activated complement components, which are released during the complement activation, have been also observed in peripheral blood of AMD patients [78–80]. Consistently, complement regulators, such as Vitronectin, Clusterin, and MCP, are highly expressed in drusen and RPE cells adjacent to drusen [30, 81, 82]. Drusen are especially characterized by Amyloid beta accumulation, which in turn is produced by senescent RPE cells and may induce oxidative stress [83]. Binding of Amyloid beta to FI results in complement activation and chronic low-grade inflammation [83]. During RPE aging, the accumulation of lipofuscin and bis-retinoid component N-retinylidene-N-retinylethanolamine has also been observed, which reduces the degradation of phospholipids by lysosomes [84, 85]. The accumulation of undigested lipids, combined with oxidative stress, leads to the formation of lipid peroxidation products [86], which in turn can induce apoptosis and complement activation [87, 88].

## 5. The Role of Common Variants in the Pathogenesis and Treatment of AMD

### 5.1. Complement Factor H (FH)

FH is produced in the liver and secreted as a protein composed of 20 short consensus repeats (SCRs), which share homology at specific residues [89, 90]. The 1q32 region, known as the regulators of complement activation (RCA) cluster, also contains five homologous CFH-related genes (*CFHR1* to* CFHR5*), encoding FH-related proteins (FHR1-5) [91]. FH is also locally produced by RPE and contributes to C3 convertase decay, preventing the amplification of C3b deposition.

In 2005, several genetic association studies, conducted by independent research groups, identified the CFH gene on chromosome 1q32 as the first gene associated with AMD risk [76, 92–94]. The most prominent effect on AMD risk was initially attributed to rs1061170 polymorphism, which leads to an amino acid change at position 402 of the FH polypeptide (Y402H). Prevalence of the 402H risk variant varies across ethnicities [95], with an increased AMD risk of 2.5 times among heterozygous individuals and 6.0 times among homozygotes [96]. This finding was confirmed by pooled analysis in both Caucasians [95] and Asians [97–99]. A more recent meta-analysis stratified by stage of disease and ethnicity, including data of 27418 AMD patients and 32843 controls, stated that the polymorphism is significantly associated with AMD: in Caucasian the mutated allele confers a 1.44 risk of early AMD, a 2.90 risk of dry AMD and a 2.46 risk of wet AMD; in Asians, the mutated allele seems to be associated only with wet AMD [100].

The rs1061170 polymorphism has been also identified as a predictor of response to anti-VEGF treatment; homozygotes individuals were less likely to achieve a better outcome than those carrying wild type genotype, suggesting the need of more effective therapeutic strategies for this subgroup of patients [101].

Conversely to this well-known genetic risk factor, the rs800292 polymorphism, a coding variant in the SCR1 domain, has been found to be protective against AMD in both Caucasians and Asians [99, 102]. This polymorphism, which leads to an amino acid change at position 62 of the FH polypeptide (V62I), also conferred a better response to treatment of neovascular AMD [101].

Besides these polymorphisms, the impact on AMD risk of other* CFH* genetic variants is still under debate. A recent meta-analysis [103] aimed to resolve inconsistent findings from studies on distinct ethnic populations about the role of four coding and noncoding variants: two noncoding variants in intron 14 (543G>A, rs1410996) and intron 15 (3144C>T, rs1329428); a coding synonymous variant in exon 10 (A473A, rs2274700); a promoter variant, positioned 257 upstream in the* CFH* promoter region (257 C>T, rs3753394). Pooled results demonstrated that these polymorphisms are significantly associated with increased AMD risk, but none of them was related to response to treatment [104].

### 5.2. Complement Component 3 (C3)

The* C3* gene, located on chromosome 19p13.3-13.2, consists of 41 exons encoding for 1663 amino acids and 13 functional domains. C3 protein is biologically inactive until it undergoes to conformational changes, which expose binding sites for pathogenic cell surface and other complement components [105]. Although several studies suggest the association between* C3* polymorphisms and AMD, findings are conflicting [106–110]. The rs2230199 polymorphism, leading to the R102G substitution, is the most commonly investigated, since it seems to influence C3 binding capacity and cofactor activity, thereby extending convertase lifetime [111]. Overall, this polymorphism was associated with AMD risk, even though this finding was confirmed in Caucasians but not in Asians [112]. A further meta-analysis confirmed the increased AMD risk associated with rs2230199 polymorphism and suggested the adverse effect of rs1047286 and rs11569536 polymorphisms on the disease [113]. By contrast, the rs2250656 polymorphism has been found to be protective against AMD [113].

Lack of evidence exists about the effect of C3 genetic variants on response to AMD treatment [114–117]. Particularly, the Comparison of AMD Treatments Trials (CATT) showed no significant effect of rs2230199 polymorphism on both visual and anatomical outcomes, after anti-VEGF therapy [118].

However, analysis of changes in central macular thickness after ranibizumab treatment, showed that the minor allele of rs2250656 SNP was associated with improvement in retinal thickness and architecture [119].

### 5.3. Factor B and C2

The* CFB* gene is located in the major histocompatibility complex (MHC) class III region on chromosome 6p21. Several lines of evidence suggest that polymorphisms in this region are associated with reduced AMD risk. Among these, pooled results from previous meta-analyses confirmed the protective effect on AMD risk of the common rs641153 polymorphism, also known as R32Q, in Caucasians [120] and in other ethnic groups [121].

The MHC class III region also includes genes encoding for proteins involved in the regulation of the immune reaction, such as* C2* gene that is located 500 bp upstream from* CFB* gene. C2 is a serum glycoprotein that functions as part of the classical pathway of the complement system. Two polymorphisms (rs9332739 and rs547154) have been directly associated with AMD by decreasing the risk of 45% and 53%, respectively [120]. However, these variants may be indirectly linked to AMD risk due to linkage disequilibrium with* CFB*. Indeed, some common haplotypes, spanning* CFB* and* C2* genes, are considered highly protective against AMD [122]. Genetic and functional studies suggest that CFB rather than C2 polymorphisms are more likely to determine the reduced AMD risk. The rs9332739 and rs547154 polymorphisms in C2 are noncoding variant, whereas the rs641153 polymorphism in CFB results in reduced alternative pathway amplification and hemolytic activity of the CFB protein [123, 124]. Moreover, after adjustment for genetic and nongenetic risk factors, the association with rs641153 proved to be robust whereas the association with rs9332739 and rs547154 became insignificant [125].

Lack of evidence exists about the effect of CFB and C2 genetic variants on response to intravitreal anti-VEGF injections; particularly, the rs641153 polymorphism did not show any pharmacogenetics effects in patients with neovascular AMD [104, 126].

### 5.4. Factor I

The CFI gene, located on chromosome 4q25, consists of 13 exons encoding for a precursor protein in hepatocytes, macrophages, lymphocytes, endothelial cells, and fibroblasts. The first eight exons encode the heavy chain, and the last five exons encode the light chain, which contains the serine protease domain. To obtain the active protein, the precursor is cleaved into heavy and light chains, which form a heterodimeric glycoprotein. This heterodimer can prevent the assembly of convertase enzymes by cleaving of C4b and C3b. The association between* CFI* polymorphisms and AMD was firstly reported by Fagerness et al. [127]. Afterwards, several studies identified polymorphisms that can alter gene expression and protein production [128–131]. The association between AMD risk and rs10033900 polymorphism is the most investigated, but results are still conflicting. To date, an updated meta-analysis showed that carriers of rs10033900 polymorphism have a reduced risk of developing AMD; these results were confirmed in Caucasians, but not in Asians [132].

## 6. The Role of Rare Variants in AMD

Growing body of evidence supports the role of rare variants, with large effect sizes, in the pathogenesis of AMD. Accordingly, targeted genomic resequencing of selected loci pointed out the effect of nonsynonymous rare variants in four complement genes (i.e., CFH, CFI, C3, and C9). These variants and their implication for personalized treatment have been recently reviewed elsewhere [102]. The CFH rs121913059 polymorphism consists of a missense mutation in the C-terminal region of the protein, which leads to an amino acid change at position 1210 of the FH polypeptide (R1210C). The R1210C variant conferred a 47-times higher risk of developing AMD [133], independently of the common rs1061170 variant. Particularly, the R1210C variant is associated with a typical phenotype with extensive drusen accumulation, as well as with earlier age of onset of the disease [134]. Whole-exome sequencing of families with AMD allowed identifying R53C and D90G variants which accelerate activity and cofactor-mediated inactivation of FH [135]. More recently, both high penetrant splice site variant (IVS6+1G>A) and coding variants (N90G, R127H, R175P, R175G, C192F, and S193L) have been proposed to explain the high burden of disease in AMD families with unknown genetic risk factors [102, 105]. Among rare variants, the K155Q variant in C3 has been independently associated with AMD [106–109], with an overall 3-fold increased risk of developing the disease [110]. In addition, Duvvari et al. [136] identified four additional genetic variants (K65Q, R161W, R735W, and S1619R) by sequencing of all coding exons of the* C3* gene; however, none of these associations was further confirmed in independent cohorts [137]. Several rare and highly penetrant* CFI* variants have been identified in patients with AMD [108]. Particularly, the majority of mutations affect the catalytic domain of the protein, leading to secretion defect and decreasing FI-mediated cleavage of C3b. Among these, van den Ven et al. demonstrated that the missense G119R substitution conferred a 22-times higher risk of AMD [138].

## 7. Interaction of Genetic Variants with Environmental Risk Factors

### 7.1. Smoking

Evidence from candidate gene studies of AMD-associated loci suggested that smoking might be an effect modifier of genetic AMD risk. Consistently with other studies [95, 139–142], results from the Beaver Dam Eye cohort did not show significant multiplicative interaction between smoking and rs1061170 polymorphism on AMD incidence and progression [143]. However, the rs1061170 polymorphism showed a stronger effect on AMD risk among smokers [139, 141, 142, 144–146]. Particularly, the Rotterdam Study reported that, among smokers, homozygosity for the risk variant conferred a 34-fold increased risk of late AMD compared to nonsmoking wild type subjects [147]. A study of discordant sibling pairs further specified that the combination between smoking more than ten pack-years and homozygosity for the risk variant was associated with a 144-fold increased risk of wet AMD, compared to nonsmoking heterozygous or wild type individuals [139]. Accordingly, the retrospective analysis of data from 385 eligible patients included in the European Genetic Database, a multicenter database for clinical and molecular analysis of AMD, demonstrated that the presence of homozygous risk variant among smokers was associated with earlier onset of wet AMD [148]. Moreover, the independent multiplicative effect of CFH genotype and smoking was more evident for some features of early AMD (i.e., central soft drusen, large area of soft drusen, and pericentral pigmentary abnormalities) associated with higher risk of AMD progression [149].

Overall, these findings indicate that smoking and rs1061170 polymorphism have independent multiplicative effects on AMD risk, with no significant interaction. The biological plausibility of this relationship might be explained by the well-known effects of smoking and CFH polymorphism on the activation of alternative pathway: on one hand, smoking alters binding of CFH to C3 and lowers plasma CFH levels [150, 151]; on the other hand, the presence of rs1061170 polymorphism alters the ability of CFH to bind to C3b.

### 7.2. Dietary Intake

In the last decades, it has been consistently demonstrated that an adequate intake of omega-3 fatty acids, lutein, zeaxanthin, and other antioxidants represents the only well-known protective factor against AMD onset and progression [19–22]. However, few studies have previously explored whether genetic susceptibility could modify this association.

While lutein and zeaxanthin supplementation clearly decreases the progression from early to advanced AMD [152], evidence on the effect of their intake through the diet is still controversial, probably due to genetic susceptibility and/or other unmeasured effect modifiers. The Rotterdam study showed a synergic biological interaction between CFH rs1061170 polymorphism and dietary intake of antioxidants, suggesting that higher intake of zinc, *ω*-3 fatty acids, *β*-carotene, lutein, and zeaxanthin might reduce the incidence of early AMD in subjects at higher genetic risk [153]. Consistently, pooled analysis of Blue Mountains Eye and Rotterdam cohorts showed that dietary intake of lutein and zeaxanthin was inversely associated with the risk of early AMD, only in concurrence with at least two risk alleles of CFH rs1061170 and ARMS2 rs10490924 polymorphisms [154]. By contrast, in absence of genetic susceptibility, higher intake of lutein and zeaxanthin was associated with greater incidence of early AMD [154]. Analysis of the Atherosclerosis Risk in Communities (ARIC) Study added to this mounting controversial evidence, demonstrating that greater lutein and zeaxanthin intake were associated with lower AMD prevalence among carriers of the heterozygous CFH genotype, higher prevalence among carriers of the homozygous risk genotype, and no statistically significant association among those with nonrisk genotype [155].

Growing body of evidence demonstrated that the anti-inflammatory and antioxidant properties of omega-3 long chain polyunsaturated fatty acids slow the progression to advanced AMD [4, 22, 156–158]. In the Age-Related Eye Disease Study (AREDS), increased intake of docosahexaenoic acid (DHA) and eicosapentaenoic acid (EPA) was associated with reduced dry AMD risk, after adjustment for behavioural factors and genetic variants, including SNPs in CFH, ARMS2/HTRA1, CFB, C2, C3, CFI, and LIPC genes [159]. In addition, the Blue Mountain Eye Study demonstrated that weekly consumption of fish was associated with lower risk of late AMD, only among subjects with the CFH homozygous risk genotype [160]. More recently, the joint effect of high-risk genotypes and vitamins intake has been also evaluated. A cross-sectional analysis of the Inter99 Eye Study suggested a significant interaction between vitamin A and rs1061170 CFH polymorphism, with a positive association between dietary intake and drusen diameter, among subjects with the homozygous risk genotype [161]. Findings from a subsample of the AREDS study also demonstrated a significant interaction between folate intake and the rs2230199 C3 polymorphism: the risk of AMD progression was lower among subjects with homozygous nonrisk genotype, but not in those carrying the risk allele. By contrast, no significant effect on AMD progression was evident for dietary intake of thiamin, riboflavin, niacin, and vitamins B6 and B12 [162]. Although foods and nutrients are consumed in combination, the abovementioned studies used single-nutrient or a single-food approach, without taking into account potential synergistic effects. To our knowledge, the study by Merle et al., including participants of the AREDS, was the first to evaluate the interaction between genetic risk factors and overall diet [163]. Particularly, the adherence to the Mediterranean diet was associated with lower risk of progression to advanced AMD among subjects with nonrisk genotype, but not among those with the homozygous risk genotype [163]. The significant association, in absence of genetic susceptibility, might be explained by the protective effect of Mediterranean diet on immune and inflammatory responses.

## 8. Interaction of Genetic Variants with AMD Treatments

The effect of the interaction between nutritional supplements and genetic susceptibility on the progression to advanced AMD is currently under debate. In 2008, for the first time, Klein and colleagues demonstrated that the effect of combined antioxidant and zinc supplementation on the progression to advanced AMD was greater among subjects with nonrisk genotype for the CFH rs1061170 polymorphism, compared with high-risk subjects [164]. Seddon and colleagues, investigating the progression to advanced AMD among subjects with low CFH and high ARMS2 genetic risk, reported that antioxidant and zinc supplementation reduced the risk of progression to wet AMD, with no significant effect on dry AMD [165]. Awh et al. first reported that zinc supplementation reduced progression to advanced AMD, among subjects with no risk alleles for CFH and at least one risk allele for ARMS2 [166]. The same research group further demonstrated a distinct effect on disease progression according to the number of risk alleles for these SNPs: supplementation with zinc, alone or as a component of the AREDS formulation, was protective against the harmful effect of the ARMS2 risk allele but it increased the risk posed by CFH allele [166]. These findings are supported by current knowledge about physiologic implication of zinc binding to CFH, which might neutralize the ability to inactivate C3 convertase [167–169]. This, together with functional consequences of CFH rs1061170 polymorphism, might cause the detrimental effect associated with concurrence of CFH risk genotypes and zinc supplementation [170]. By contrast, data analysis of a larger AREDS subsample found no interaction between AREDS formulation and genetic susceptibility [171]. However, the design of this study does not allow us to exclude if the absence of interaction was caused by underpowered statistical analysis.

While the AREDS formulation may slow the progression to dry AMD by modulating complement activity [172], intravitreal injections of anti-VEGF agents are currently considered part of the standard treatment regimen for neovascular AMD, accompanied by photodynamic therapy (PDT) with verteporfin. In spite of the well-established effect of CFH rs1061170 polymorphism on AMD risk, there is still controversy about its role in the response to anti-VEGF treatment. To our knowledge, Chen et al. were the first to summarize data on the relationship between the rs1061170 polymorphism and response to treatment of neovascular AMD [32]. Pooled analysis indicated that CFH risk genotypes were weakly but significantly associated with less effective response to any form of treatment, including anti-VEGF agents, photodynamic therapy, and antioxidants/zinc supplementation [32]. This finding was further confirmed by more specific meta-analyses of studies, investigating the relationship between CFH rs1061170 polymorphism and response to anti-VEGF treatment [97, 173].

In summary, evidence on the interaction between genetic susceptibility and response to AMD treatment is currently weak and controversial, raising the need of further researches prior to applying genetic testing to personalized medicine.

## 9. Implications for Preventive and Personalized Medicine

Uncovering the interaction between genome and environment is one of the main challenge towards preventive and personalized medicine. The discovery of genetic variants in genes for complement proteins pointed out the role of chronic inflammation and complement regulation in AMD pathogenesis. While the effect of common and rare genetic variants is well established, our review suggests that environmental exposure could modulate the genetic-associated risk of onset and progression of AMD, as well as therapeutic response.

Since the identification of high-risk patients can improve clinical management of AMD, several prediction models of onset and progression are now widely available [174, 175]. These models, based on a small number of common genetic variants, are suitable to distinguish subjects who will and will not suffer from AMD, with an area under the curve that ranges between 0.8 and 0.9 [174, 175]. However, the evaluation of these models did not provide encouraging results, because the same subject can receive controversial forecasts from different tests [176, 177].

To date, it is difficult to evaluate the benefits of genetic testing in the context of complex diseases such as AMD [178]. To overcome this issue, prediction models should also include rare mutations, like those reviewed by Geerlings et al. [102], clinical characteristics, and environmental risk factors. Once early AMD is clinically manifested, the number and nature of risk alleles significantly influence the progression to advanced AMD. Moreover, in addition to independent risk factors (i.e., smoking) [95, 139–143], others, such as diet [163] and nutrients intake [153, 154], seem to interact with AMD-associated polymorphisms on determining the risk of progression to advanced AMD. Growing body of evidence also suggested determining the genetic risk profile prior to choosing the adequate treatment. In this context, we concluded that success of treatment of dry AMD with antioxidants and zinc relies on genetic risk variants, with a better response among subjects with no CFH risk alleles [164–166]. Similarly, the presence of CFH risk genotypes leads to worse response to anti-VEGF therapy against wet AMD [97, 173]. Despite the fact that knowledge is increasing, the perspective to guide personalized medicine through genetic testing is still under debate and further clinical studies should be encouraged.

Several lines of evidence also suggested that complement system is a promising target for the development of novel therapies, which could support the conventional treatment with anti-VEGF agents. Currently, potential candidates, such as complement component inhibitors, antibody-based compounds, and receptor antagonists, are in clinical trials or in preclinical evaluation [179]. While eculizumab, a humanized IgG antibody against complement component 5 (C5), seems to be ineffective in the management of dry AMD patients [180], treatment with lampalizumab, an antibody that inhibits complement factor D, reduced the progression of geographic atrophy lesion [181]. Since treatment with lampalizumab seems to be more effective in patients with specific CFI genotypes, a phase III trial is currently running. In this perspective, understanding the pathways involved in inflammation and neovascularization could allow the choice of proper treatment within the clinical context of disease heterogeneity.

In conclusion, our review highlighted that research behind the role of complement system in AMD has been mainly based on genome-wide and candidate gene studies. However, genomics alone does not reveal the causative relation between gene-environment interaction and AMD, and current evidence should be integrated by other “omics” disciplines which take into account the impact of exposome. However, in the forthcoming future, it is plausible that AMD prevention and treatment will be personalized for single groups of patients, according to their genetic risk profile, clinical characteristics, and environmental exposure.
